# Retraction: Recombinant Human Adenovirus-p53 Injection Induced Apoptosis in Hepatocellular Carcinoma Cell Lines Mediated by p53-Fbxw7 Pathway, Which Controls c-Myc and Cyclin E

**DOI:** 10.1371/journal.pone.0231287

**Published:** 2020-03-27

**Authors:** The PLOS ONE Editors

After this article [1] was published, concerns were raised about the mouse tumor sizes reported in Figure 5.

The underlying data for Figure 5, which were provided in post-publication discussions, confirmed that tumors up to 8.6 cm^3^ were observed in these experiments. The authors also confirmed that in some cases tumors exceeded 20 mm in diameter. They provided general information about health monitoring and endpoint guidance at their institution, and noted that animal health was monitored every three days with increased monitoring of clinical signs and symptoms for animals bearing tumors > 1.5 cm in diameter. In response to queries about the experimental endpoints, the authors clarified that they planned the experiments aiming to assess differences in tumor growth and survival, but at five weeks post-implantation they terminated the experiment because they observed three cases in which tumors impaired locomotion. The authors did not provide further study-specific information as to humane endpoint criteria or specific animal welfare considerations.

A member of *PLOS ONE*’s Animal Research Advisory Group assessed the article and the authors’ comments and confirmed that the tumor sizes reported in this article far exceed community standards for humane endpoint limits in mouse tumor studies. The advisor noted that the tumor results present a significant animal welfare and ethical concern.

In light of the above concerns, the *PLOS ONE* Editors retract this article. The editors regret that these concerns were not addressed at the time of the original review process.

KT notified the journal that all authors disagree with retraction. XZ, ZZ, CL, JZ, JG, YY, QL either could not be reached or did not respond directly.
